# Effects of Extracellular Vesicles from Blood-Derived Products on Osteoarthritic Chondrocytes within an Inflammation Model

**DOI:** 10.3390/ijms22137224

**Published:** 2021-07-05

**Authors:** Alexander Otahal, Karina Kramer, Olga Kuten-Pella, Lukas B. Moser, Markus Neubauer, Zsombor Lacza, Stefan Nehrer, Andrea De Luna

**Affiliations:** 1 Center for Regenerative Medicine, Danube University Krems, 3500 Krems, Austria; karina.kramer@donau-uni.ac.at (K.K.); lukas.moser@donau-uni.ac.at (L.B.M.); markus.neubauer@donau-uni.ac.at (M.N.); stefan.nehrer@donau-uni.ac.at (S.N.); andrea.deluna@donau-uni.ac.at (A.D.L.); 2 OrthoSera GmbH, 3500 Krems, Austria; olga.kuten@orthosera.com (O.K.-P.); zsombor.lacza@orthosera.com (Z.L.); 3 Institute of Sport and Health Sciences, University of Physical Education, 1123 Budapest, Hungary

**Keywords:** extracellular vesicles, inflammation, immunomodulation, coagulation, intercellular communication, blood products, osteoarthritis

## Abstract

Osteoarthritis (OA) is hallmarked by a progressive degradation of articular cartilage. One major driver of OA is inflammation, in which cytokines such as IL-6, TNF-α and IL-1β are secreted by activated chondrocytes, as well as synovial cells—including macrophages. Intra-articular injection of blood products—such as citrate-anticoagulated plasma (CPRP), hyperacute serum (hypACT), and extracellular vesicles (EVs) isolated from blood products—is gaining increasing importance in regenerative medicine for the treatment of OA. A co-culture system of primary OA chondrocytes and activated M1 macrophages was developed to model an OA joint in order to observe the effects of EVs in modulating the inflammatory environment. Primary OA chondrocytes were obtained from patients undergoing total knee replacement. Primary monocytes obtained from voluntary healthy donors and the monocytic cell line THP-1 were differentiated and activated into proinflammatory M1 macrophages. EVs were isolated by ultracentrifugation and characterized by nanoparticle tracking analysis and Western blot. Gene expression analysis of chondrocytes by RT-qPCR revealed increased type II collagen expression, while cytokine profiling via ELISA showed lower TNF-α and IL-1β levels associated with EV treatment. In conclusion, the inflammation model provides an accessible tool to investigate the effects of blood products and EVs in the inflammatory context of OA.

## 1. Introduction

The degenerative disease osteoarthritis (OA) is one of the leading causes of disability, especially in elderly people, affecting 10% of men and 18% of women above 60 years of age [1]. Due to its increasing incidence, OA is becoming a major health problem, especially in the Western world. Risk factors favoring the onset of OA include obesity, genetic predisposition, trauma, muscle weakness, physical activity levels, bone density, and nutritional status [2,3]. It is hallmarked by cartilage damage, intraarticular synovitis, subchondral remodeling, and joint pain [4]. In healthy cartilage, a balance between anabolic and catabolic processes occurs [5,6], and this balance is disrupted in OA cartilage, favoring catabolic, matrix-degrading events. As a whole-joint disease, OA’s pathophysiology involves not only cartilage degradation, but also synovitis, subchondral bone remodeling, meniscal degeneration, and infrapatellar fat pad inflammation and fibrosis [7,8]. For the most part, these processes are mediated by the two proinflammatory cytokines interleukin 1β (IL-1β) and tumor necrosis factor α (TNF-α), produced mainly by chondrocytes, synovial fibroblasts, and macrophages [9]. On the one hand, IL-1β and TNF-α can induce the expression of other proinflammatory cytokines—such as IL-6, IL-15, IL-17, and IL-18 [10,11,12,13]—and on the other hand, they promote the production of the matrix-degrading enzymes matrix metalloproteinases (MMPs)—MMP-1, MMP-3, MMP-13—as well as a disintegrin and metalloproteinase (ADAM) with thrombospondin-1 domains (ADAMTS)-4 and -5. These induce the destruction of the matrix proteins type II collagen, glycosaminoglycans, and proteoglycans [14,15,16].

Depending on the disease stage, conventional treatment of OA involves pharmacological and non-pharmacological options, cell-based therapies, or total knee replacement as a last resort [17,18,19]. The Kellgren–Lawrence system classifies OA into 5 groups—grades 0–4—depending on the radiographic appearance of the disease, such as thickness of the remaining cartilage, joint space narrowing, or osteophyte formation [20]. While pharmacologic and non-pharmacological treatments are indicated for low-grade OA, patients with severe OA undergo total knee replacement surgery [21,22]. Currently, these treatment approaches are mostly palliative rather than curative, meaning that the patient is relieved from pain and inflammation, but the cartilage and, subsequently, its mechanical function is not restored. Therefore, new treatment options are becoming the focus of regenerative medicine research. Blood derivatives such as platelet-rich plasma (PRP) are widely used in orthopedics, wound healing, and dentistry [23,24,25,26], with promising outcomes regarding the mediation of cell migration and proliferation, as well as of anti-inflammatory signals [27,28]. The principle of PRP lies in the high amount of platelets and subsequently high concentration of growth factors within a small volume of plasma—especially transforming growth factor β (TGF-β), platelet-derived growth factor (PDGF), and platelet factor 4 (PF4) [29]. Upon injection of PRP into the diseased area, these growth factors stimulate tissue homeostasis in the joint. They promote extracellular matrix synthesis [30,31], hyaluronic acid release from the synovium [32], and inhibition of inflammation driven by IL-1β and TNF-α [33]. Improved clinical scores were reported in response to PRP treatment of knee osteoarthritis [34]. Drawbacks connected with the application of PRP include high donor variations and the availability of over 20 different devices on the market, which all produce different PRPs in the context of cellular content and growth factor profiles [35,36]. Therefore, the feasibility of PRP standardization efforts is restricted. To circumvent these drawbacks, cell-free alternatives must be established. Hyperacute (hypACT) serum is a potential alternative to PRP; per definition, it is devoid of cells, and consists of serum from a platelet-rich fibrin clot, which is essentially the serum harvested at a hyperacute phase [37]. Within this clot, platelets are trapped, but secrete their content into the serum. In theory, upon intra-articular injection, hypACT is considered to trigger similar processes that occur during injury, whereby a fibrin clot is formed in order to stimulate wound healing and tissue homeostasis [38]. The effects of hypACT in the context of OA were investigated in a co-culture study involving cultures of cartilage, subchondral bone, and synovial membrane explanted from osteoarthritic knees in the presence of IL-1β for 2 days [9]. Afterwards, the medium was replaced with hypACT-supplemented medium, and 39 biomarkers were evaluated in the co-culture supernatant. HypACT treatment resulted in decreased levels of the proinflammatory markers IL-1β, TNF-α, and IL-6 receptor α (IL-6Rα), as well as in the induction of IL-1 antagonists (IL-1RA), and cell viability. However, the mode of action of plasma- and serum-based blood products remains unclear. Most studies focus on evaluating the growth factors within the respective blood derivatives, although other components—such as extracellular vesicles (EVs)—should be taken into consideration. EVs are nanoparticles ranging from 30 to 5000 nm and are surrounded by a lipid bilayer. As size ranges overlap, they are categorized based on their site of origin. Exosomes are generated inside multivesicular bodies, and are released via exocytosis, while biosynthesis of microvesicles occurs at the cell surface via budding and apoptotic bodies, which are membrane blebs of dying cells [39,40]. EVs can be found in all body fluids—including blood, plasma, urine, saliva, amniotic fluid, and synovial fluid [41,42]—and they can transport bioactive molecules (mRNA, miRNA, proteins, lipids, enzymes, and DNA fragments) from one cell to another [43]. The application of EVs for cartilage regeneration has been extensively investigated both *in vitro* and *in vivo*, and their role in inflammatory arthritis has already been demonstrated (reviewed in [1,44,45]). The majority of these EVs were isolated from cell culture supernatants of mesenchymal stromal cells. Less is known about the role of EVs isolated from blood derivatives in cartilage regeneration. Therefore, we aimed to investigate the role of EVs isolated from PRP and hypACT in an inflammation model, in which patient-derived osteoarthritic chondrocytes were co-cultured with primary activated proinflammatory M1 macrophages. We compared these EVs with the respective blood products from which they were isolated, in order to determine to what extent blood-derived EVs were able to modulate proinflammatory cytokine secretion and to stimulate expression of cartilage genes in diseased chondrocytes. The rationale of setting up a co-culture model involving primary chondrocytes exposed to activated macrophages was to mimic the inflammatory environment in an OA joint. The response of this model to EV treatment was considered to represent a more physiological and reliable readout than observations from conventional 2D culture of chondrocytes in isolation. Results from the co-culture model will help to better understand which components of the investigated blood products might be the mediators of their regenerative potential.

## 2. Results

**Figure 1:**
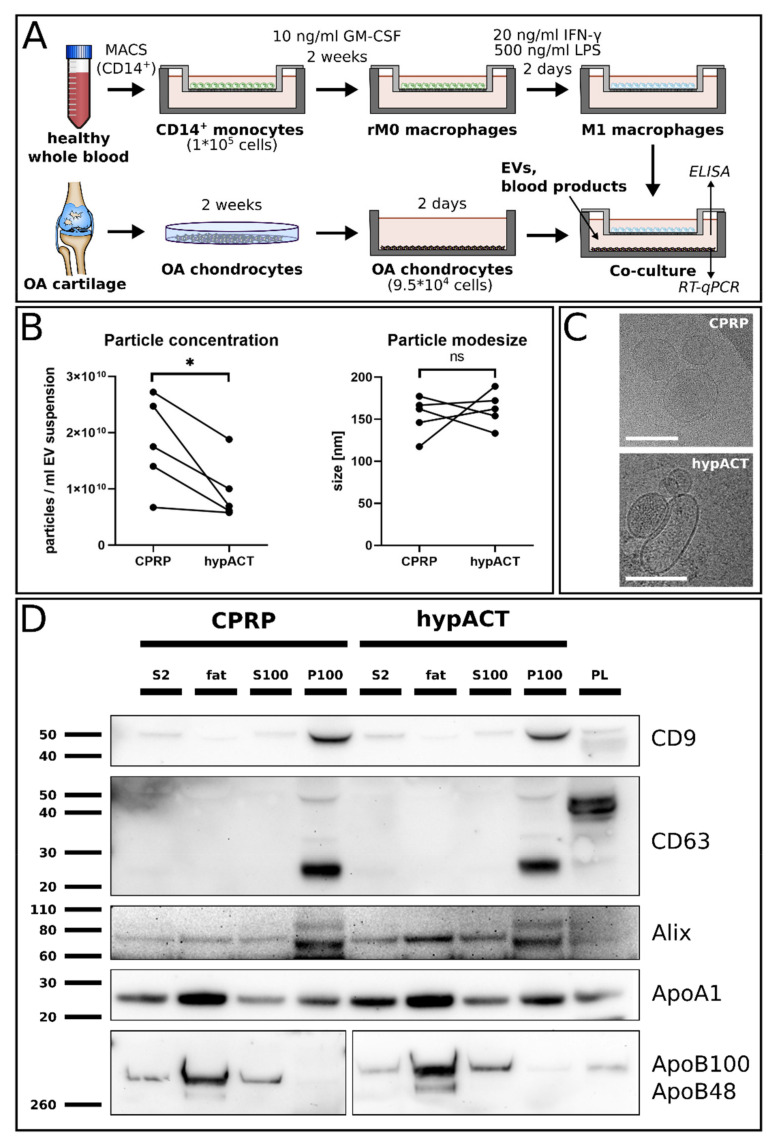
(**A**) Schematic overview of the procedure to set up the co-culture model. (**B**) Characterization of the concentration and mode size of EVs enriched via UC, analyzed via NTA. (**C**) Visualization of EVs via cryo-electron microscopy (scale bars: 200 nm). (**D**) Protein profiling of enriched EVs screening for positive EV markers CD9, CD63, and Alix, as well as the negative markers ApoA1 and ApoB100/48. S2: precleared blood product as input material; fat: buoyant layer after UC; S100: supernatant after UC; P100: pellet after UC; PL: platelet lysate as positive control; *: *p* < 0.05; ns: not significant.

Prior to setting up the co-culture system schematically presented in Figure 1A, EVs were enriched via ultracentrifugation (UC) from the blood products CPRP and hypACT. Particle concentrations and particle mode sizes were determined via NTA in resuspended EV pellets after UC (Figure 1B). While mode sizes were similar between EVs from CPRP and hypACT, higher concentrations of EVs were obtained from CPRP compared to hypACT (t(4) = 3.157; *p* = 0.0343). Cryo-electron microscopy was performed to visualize EVs enriched from CPRP and hypACT (Figure 1C). Western blot analysis (Figure 1D) confirmed the enrichment of EV markers CD9, CD63, and Alix in P100 fractions. CD63 appeared as bands of different molecular weight in P100 fractions compared to platelet lysate, which was used as a positive control. This might result from post-translational modifications such as glycosylation or ubiquitination, suggesting an association of unprocessed CD63 with EVs [44,45]. EVs were devoid of buoyant lipoproteins, indicated by the absence of ApoB100/48, which is found in (V)LDL particles. However, pelleted EVs contained HDL particles, as ApoA1 was detected in the P100 fractions. The investigated EV biomarkers do not allow the differentiation of exosomes and microvesicles enriched via ultracentrifugation, as there is currently no marker that would uniquely identify an EV as an exosome or microvesicle after being released from a cell [46]. A distinction of EV subtypes was not intended, because the study aimed to investigate the biological effects of the whole population of EVs in the blood products.

To verify the successful enrichment of CD14^+^ monocytes from the whole blood of healthy donors, cells were stained with PE-labeled antibody directed against CD14 and analyzed via flow cytometry. As presented in Figure S1, CD14+ cell populations were assessed in the eluate, the flowthrough, and the input material before magnetic-activated cell sorting (MACS). The input showed a small CD14^+^ population, which was absent in the flowthrough. The eluate contained cells highly enriched for CD14 (Figure S1A). A direct comparison of the abundance of CD14-positive and CD14-negative cells in the three fractions is shown in Figure S1B.

**Figure 2:**
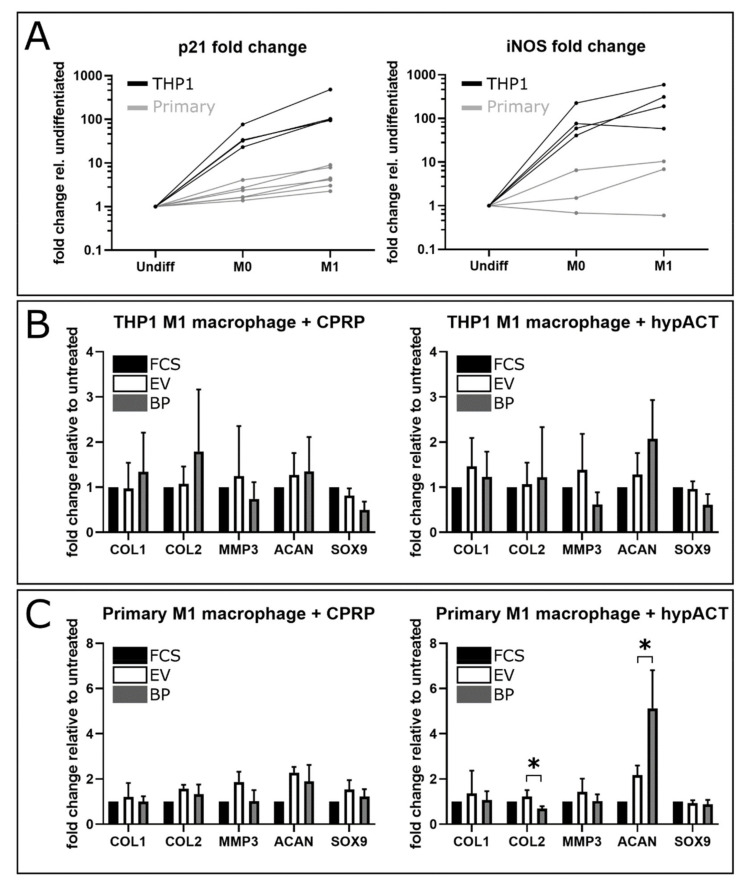
(**A**) Verification of macrophage activation via determining the increase in p21 and iNOS gene expression from undifferentiated monocytes to M1 macrophages. Data are given from 4 (THP1) or up to 6 (primary) experiments. iNOS fold change shows only 3 samples, because no iNOS was detected in undifferentiated primary monocytes from 3 donors. (**B**,**C**) Gene expression analysis of OA chondrocytes in co-culture with THP1 M1 macrophages (**B**) or primary M1 macrophages (**C**). Data are given as fold change normalized to FCS-supplemented co-cultures from 4 individual chondrocyte donors measured in triplicates ± SD. FCS: fetal calf serum supplementation; EV: supplementation with extracellular vesicles; BP: supplementation with blood products. *: *p* < 0.05.

To confirm the differentiation of monocytes into macrophages, expression levels of p21 and inducible nitric oxide synthase (iNOS) were assessed via RT-qPCR (Figure 2A). These genes have been identified as markers of monocyte differentiation [47,48]. Undifferentiated monocytes did not show measurable iNOS expression in three out of six donors. Primary monocytes from one male and two female donors expressed iNOS; therefore, only data from these donors are shown in Figure 2A, as the expression levels of iNOS are normalized to the iNOS expression in primary monocytes.

### 2.1. Gene Expression Changes in Response to Blood Product or EV Supplementation

To determine gene expression changes in response to blood product or enriched EV treatment in patient-derived OA chondrocytes in co-culture with primary M1 macrophages or THP1 cells, the expression levels of type I collagen (COL1), type II collagen (COL2), matrix metalloproteinase 3 (MMP3), aggrecan (ACAN), and SRY-box transcription factor 9 (SOX9) were analyzed via reverse transcription quantitative PCR (RT-qPCR) (Figure 2B,C). Expression levels were normalized to chondrocytes cultured in the presence of FCS, in order to compare expression changes in response to CPRP or hypACT blood products or EVs. Primary chondrocytes did not significantly alter the expression of COL1, COL2, or MMP3 in the presence of either blood products or EVs after 48 h co-cultured with THP1 cells or primary M1 macrophages, as shown by two-way ANOVA and Tukey’s post-hoc test. Supplementation with hypACT blood product strongly increased ACAN expression in chondrocytes in co-culture with primary M1 macrophages (Figure 2C). ACAN expression was significantly higher compared to hypACT EVs (F(4,30) = 8.065, *p* < 0.0001) and to CPRP blood products (F(4,30) = 8.801, *p* < 0.0001) or CPRP EV supplemented co-cultures (F(4,30) = 7.745, *p* < 0.0001). Comparing SOX9 expression in the presence of hypACT or CPRP EVs revealed elevated SOX9 levels (F(4,30) = 5.476, *p* = 0.0046) driven by CPRP EVs in the presence of primary M1 macrophages. SOX9 expression was also higher in response to CPRP EVs than to hypACT blood products (F(4,30) = 6.051, *p* = 0.0015), but not compared to CPRP blood products.

To assess differences between blood product and EV treatment within groups, data were tested with multiple *t*-test. Again, THP1 co-culture experiments did not yield significant differences (Figure 2B); however, primary M1 macrophage co-culture supplemented with hypACT (Figure 2C) showed a stronger expression of COL2 in the presence of hypACT EVs compared to hypACT blood products (t(6) = 3.550, *p* = 0.012), in addition to elevated expression of ACAN in response to hypACT blood products compared to hypACT EVs (t(6) = 3.406, *p* = 0.014). Reported *p*-values are FDR corrected.

### 2.2. EV Supplementation Modulates Cytokine Levels

**Figure 3:**
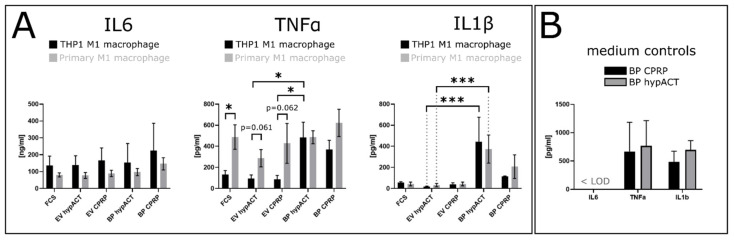
(**A**) Cytokine profiling involving IL-6, TNF-α, and IL-1β levels in the presence of THP1 or primary M1 macrophages, and in different treatments, as determined via ELISA in sandwich format. (**B**) Control media were measured before starting the co-culture. Data are from at least 4 experiments ± SD. *: *p* < 0.05; ***: *p* < 0.001.

To monitor changes in cytokine levels in response to EV or blood product supplementation in the co-culture as readouts for pro- or anti-inflammatory effects, IL-6, TNF, and IL-1β concentrations were determined in conditioned cell culture medium via enzyme-linked immunosorbent assay (ELISA) in sandwich format (Figure 3). IL-6 levels showed no significant differences—neither between nor within treatment groups—although there was a trend across treatment groups towards lower IL-6 levels in primary compared to THP1 M1 macrophage co-culture (F(1,52) = 3.682; *p* = 0.0605). TNF-α levels responded differently to EV treatment compared to blood product (BP) treatment in THP1 M1 macrophage co-culture (F(5,30) = 4.129; *p* = 0.0057). TNF-α was higher in BP hypACT than in EV hypACT (*q* = 4.453; *p* = 0.0391), as well as EV CPRP (*q* = 4.513; *p* = 0.0354) in the presence of THP1 M1 macrophages. Similarly, BP CPRP resulted in higher TNF-α levels compared to EV CPRP, although not significantly (*q* = 3.201; *p* = 0.24). In contrast, EV and BP treatment in primary M1 macrophage co-culture did not give rise to significant changes of TNF-α levels—neither for CPRP nor hypACT. Nevertheless, TNF-α levels differed between THP1 and primary M1 co-cultures in the presence of FCS, EV CPRP (*p* = 0.061), and EV hypACT (*p* = 0.062) supplementation. IL-1β levels were not significantly different within treatment groups; however, BP hypACT strongly promoted IL-1β release (F(5.27) = 11.74; *p* < 0.0001) in THP1 co-culture (*q* = 7.713; *p* = 0.0001) and primary M1 macrophage co-culture (*q* = 5.199; *p* = 0.0119) compared to EV hypACT treatment. Although there were higher levels of IL-1β present in BP-CPRP- compared to EV-CPRP-supplemented co-cultures, this difference was not significant. In addition, more IL-1β was detected for BP hypACT treatment compared to BP CPRP (*q* = 4.977; *p* = 0.0174) in THP1 co-culture. This relationship was present in primary M1 co-culture as well, although it was not significant (*q* = 2.782; *p* = 0.3859). To estimate which concentrations of cytokines are introduced into the model via blood product supplementation, cytokine levels were determined in culture media prior to addition into the co-cultures (Figure 3B). While IL-6 levels were below the limit of detection, levels of TNF-α and IL-1β were not significantly different in the control media compared to co-culture supernatant after 48 h. Taken together, IL-6 was not different between treatments, and TNF-α and IL-1β levels were either affected by the type of M1 macrophage co-culture and type of supplement, or only type of supplement, respectively.

## 3. Discussion

The co-culture of OA chondrocytes with activated macrophages revealed that the release of matrix metalloproteinases and proinflammatory mediators is exacerbated when compared with an OA chondrocyte monoculture [49]. Therefore, such an *in vitro* approach mimicking the inflammatory environment in an OA joint allows testing of pharmacological or biological agents via simple supplementation of culture media. This study established a co-culture system involving patient-derived OA chondrocytes and primary activated M1 macrophages from healthy donors put into contact in a transwell culture (Figure 1A). The co-culture was performed without hydrogel embedding of cells, in order to avoid potential barrier effects caused by the hydrogel that might hinder the access of EVs to cells. However, this might not be an issue if the inflammation model is used to test pharmacological rather than biological agents, such as blood products or blood product-derived EVs.

Previously, treatment of OA chondrocytes with blood-product-derived EVs in 2D culture resulted in elevated chondrotypic gene expression of aggrecan (ACAN), type II collagen (COL2A1), and SRY-box transcription factor 9 (SOX9) in the presence of EVs, compared to the respective blood product [50]. However, a 2D culture provides limited capability of modeling joint inflammation, although earlier studies used IL-1β supplementation to simulate an inflammatory environment [51,52,53]. Aside from IL-1β, the secretome in an OA joint also contains specific EVs released from activated M1 macrophages, for example [54,55,56]. Therefore, the aim was to extend a simple 2D culture of chondrocytes with proinflammatory stimuli elicited by activated M1 macrophages in co-culture. This meant we could build a more physiological model in order to test whether and to what extent EVs present in blood products are the basis for effects mediated by blood products. EVs were characterized via nanoparticle tracking analysis (NTA), cryo-electron microscopy and Western blot after enrichment via ultracentrifugation (Figure 1B-D), which is commonly used to enrich EVs from various biological fluids [57]. An advantage is the depletion of low-density lipoprotein (LDL) in the process (Figure 1C), as oxidized LDL (ox-LDL) is a ligand for Toll-like receptor 4 (TLR), and could counteract the beneficial effects of EVs via proinflammatory TLR4 signaling [58,59,60]. At the same time, investigating EVs isolated using just one method is a limitation of the study, as the isolation method might have an impact on the biological properties of the EVs. While UC enriches EVs with a plethora of adsorbed molecules, the extravesicular “protein corona” is different on EVs isolated by other procedures, such as those involving size-exclusion chromatography, for example [61,62,63].

Gene expression changes of COL1, COL2, MMP3, ACAN, and SOX9 in response to EVs and blood products were analyzed via RT-qPCR in primary OA chondrocytes and compared to FCS supplemented co-cultures (Figure 2A). Unfortunately, gene expression changes were limited, and significant changes were only found in primary M1 macrophage co-culture. This could either mean that neither blood product nor EV treatment resulted in substantial differential gene expression, or that EVs were sufficient to reproduce the gene expression changes mediated by blood product treatments, highlighting the essential contribution of EVs to the mechanisms of action of blood products.

Cytokine analysis was performed in this study, with the intention of uncovering effects mediated by EVs in modulating the inflammatory environment of OA. Similar to a previous work by our group [50], BP hypACT gave rise to IL-6 release in the co-culture system, while IL-6 levels in the control medium were below the limit of detection (Figure 3A,B). However, the IL-6 release was probably not a result of the type of supplement present, as no differences were found between treatments. The IL-6 release may result from the co-culture itself, as crosstalk of OA chondrocytes and synovial fibroblasts increased IL-6 release [64]. Therefore, a similar mechanism might be involved between OA chondrocytes in co-culture with activated macrophages. While TNF-α and IL-1β in BP-supplemented co-cultures were not different to the control medium, EV-supplemented co-cultures showed low levels of these cytokines (Figure 3A,B). This could mean either that EVs introduce very low levels of cytokines into the system, which might adhere to EV surfaces, or that the presence of EVs at the tested concentration was too low to achieve the expected reductions of IL-6, TNF-α, and IL-1β concentrations compared to FCS-supplemented controls. Only one EV dose was tested in the study, because the rationale was to expose cells to the same number of EVs that are present in the equivalent volume of blood product used as supplement in parallel. A dose–response study might shed light on EV-mediated effects in a more detailed way. Clearly, the low sample size is another limitation of the study, which was conducted as pilot study rather than as a clinical study evaluating the effect size of an EV-based therapy. Nevertheless, the results indicate that EV treatment is associated with lower proinflammatory cytokine levels compared to blood product treatment. In addition, the data suggest the investigation of more markers and processes within the inflammation model—such as signaling pathways involved in chondrocyte differentiation including TGF-β and Wnt signaling [65,66]—to elucidate how blood-product-derived EVs might integrate into cartilage regeneration. Finally, future studies might employ the described co-culture inflammation model to investigate the effects of EVs from other sources than blood on the inflammatory environment of an OA joint.

## 4. Materials and Methods

### 4.1. Preparation of PRP and hypACT

A volume of 40 mL of whole blood was collected in-house from 5–7 donors chosen randomly from a pool of volunteers after informed consent was signed. Blood collection was approved by the local Ethics Committee of Danube University Krems (approval date: Jan 14^th^ 2013). Inclusion criteria for blood donation were an age of between 25 and 45, as well as being in good health on the day of blood donation. This was determined using an evaluation form, whereby conditions such as pregnancy, underweight, or diabetes were deemed exclusion criteria. Citrate-anticoagulated PRP (CPRP) was generated via collecting whole blood into citrate-coated vacutainer tubes (VACUETTE 9NC trisodium citrate 3.2%, Greiner BioOne, Kremsmunster, Austria, #455322) and further processed as described in [67]. hypACT was prepared by using hypACT inject developed by OrthoSera GmbH (Krems, Austria). Within this device, blood was collected and centrifuged immediately at 1710× *g* for 5 min at room temperature (RT). As no anticoagulants were present, a fibrin clot formed inside the device, whose content was extracted by pressing the pistil into the syringe in order to obtain hypACT.

### 4.2. Enrichment of Extracellular Vesicles

We transferred 2.5 mL of freshly prepared blood products into 15-mL polypropylene tubes (TPP, #91015) and centrifuged them at 2500 × *g* for 15 min at RT in order to remove cells and cellular debris. Aliquots of the precleared supernatant (S2 fraction) were stored for protein analysis. Aliquots of 2.5 mL of cleared CPRP or hypACT were diluted 1:1 with PBS (GIBCO, Waltham, USA, #70011-036) free from Ca^2+^, and Mg^2+^ filtered through 0.22-μm sterile filters (Sartorius, Göttingen, Germany, #16534), transferred into ultracentrifugation tubes (Beckman Coulter, #355647) and centrifuged at 100,000 × *g* for 120 min at 4 °C in a MLA-80 fixed-angle rotor (k-factor 29; 45,000 rpm). The obtained pellets (P100) were resuspended in 200 μL of PBS, and aliquots of the supernatant obtained after ultracentrifugation (S100)—as well as the buoyant fat layer (fat fraction)—were stored at –80 °C after determination of whole protein concentration of aliquots lysed in RIPA buffer (Thermo Fisher Scientific, Waltham, USA, # 89900) via DC protein assay (BioRad, Hercules, USA, # 5000111), according to the manufacturer’s protocol. All relevant data were submitted to the EV-TRACK knowledgebase (EV200024) [68].

### 4.3. Nanoparticle Tracking Analysis (NTA)

To determine the size and concentration of isolated particles, NTA (ZetaView, Particlemetrix) in scatter mode was performed as previously described [69,70]. Briefly, samples were diluted 1:1000, or as required, in PBS, and pre-acquisition camera settings were kept constant at sensitivity 80 and shutter 100. Videos were made at 11 positions each with 30 frames per second for 2 s in one acquisition cycle. ZetaView 8.04.02 software was applied to analyze videos using post-acquisition parameters of 20 minimum brightness, minimum and maximum area of 5 and 1000, respectively, and 64 classes per decade. Mode particle size was determined from smoothed particle size distributions in a post-processing approach of ZetaView data files via a custom PHP implementation of the Savitzky–Golay algorithm, as described earlier [71]. Mode particle size was investigated as a readout parameter, because EV preparations show skewed particle size distributions. Therefore, arithmetic mean or median particle size could be of limited use to describe the particle size of an EV population.

### 4.4. Cryo-Electron Microscopy

Aliquots (4 μL) of EV suspensions were placed on 1-μm mesh copper grids and blotted for 1 or 2 s, before plunge-freezing in liquid ethane. Samples were visualized on a Glacios cryo-electron microscope.

### 4.5. Western Blot Analysis

Ten micrograms of total protein of EV suspension were loaded on a 4–12% SDS-PAGE Gel (Invitrogen, Waltham, MA, USA, #NP0322) under reducing (Alix, ApoB100) or non-reducing (CD9, CD63, ApoA1) conditions in the presence of 100 mM dithiothreitol. Primary antibodies against Alix (Cell Signaling, Leiden, Netherlands, #2171), CD9 (System Bioscience, Palo Alto, USA, #EXOAB-CD9A-1), and CD63 (BioLegend, San Diego, CA, USA, #353005, clone H5C9) were used to screen for EV biomarkers, while ApoA1 (Santa Cruz, Heidelberg, Germany, #sc-376818) and ApoB100/48 (Santa Cruz, #sc-393636) were detected to monitor the depletion or co-enrichment of lipoproteins. Each antibody was used diluted 1:1000 in 1% BSA in PBST (PBS + 0.1% Tween 20). HRP-conjugated anti-rabbit (Biorad, Hercules, CA, USA, #170-5046) and anti-mouse (Biorad, #170-5047) were used as secondary antibodies. Detection was performed via enhanced chemiluminescence (ECL) using WesternBright ECL substrate (Advansta, San Jose, USA, #K-12045-D20) on a ChemiDoc device (BioRad). Digital images were automatically white-corrected via the GIMP v2.8.

### 4.6. Isolation of Patient-Derived Osteoarthritic (OA) Chondrocytes

Human articular cartilage was obtained from 1 male and 5 female patients undergoing knee joint replacement surgery at the Universitätsklinikum Krems. The mean age of the donors was 68.50 ± 10.37 (SD) years, and their mean body mass index (BMI) ranged around 32.21 ± 8.64 (SD), with a mean weight of 80.76 ± 18.82 kg (SD) and a mean height of 159.0 ± 5.2 cm (SD). Informed consent was obtained from the patients, and the Ethics Committee of Lower Austria approved the study (GS1-EK-4/480-2017, approval date: 11 January 2018). Chondrocytes were isolated as previously described [72]; in brief, cartilage was cut from femoral condyles and minced into 2–3-mm^3^ pieces. After digestion with 0.2 WU/mL Liberase (Roche, Vienna, Austria, #05401119001) in DMEM-F12 GlutaMAX medium (GIBCO, Waltham, MA, USA, #11524436) supplemented with 200 U/mL penicillin, 0.2 mg/mL streptomycin, and 0.5 μg/mL amphotericin B 2 (all from Sigma-Aldrich Chemie GmbH, St. Louis, MI, USA), the cell suspension was filtered through a 40-μm cell strainer (BD Biosciences, Franklin Lakes, NJ, USA, # 352340) to remove undigested material, before seeding cells at a density of 10^4^ cells/cm^2^ in cell culture flasks in the medium described above, supplemented with 10% FCS.

### 4.7. Isolation of CD14^+^ Primary Monocytes

Blood from 6 donors (4 female, 2 male) was collected in citrate-coated vacutainer tubes (VACUETTE 9NC trisodium citrate 3.2%, Greiner BioOne, #455322), diluted 1:2 with PBS, and pipetted on a layer of Ficoll (GE healthcare, Tiefenbach, Austria, #17-1440-02). After centrifugation at 200× *g* for 40 min at RT, the PBMC layer was removed and washed with PBS. The suspension was centrifuged again at 20× *g* for 10 min at RT, and the pellet was resuspended in 5 mL DMEM-F12 GlutaMAX medium (Gibco, #11524436) supplemented with 10% FCS, 200 U/mL penicillin, 0.2 mg/mL streptomycin, and 2.5 μg/mL amphotericin B (all from Sigma-Aldrich Chemie GmbH). Cells were counted via trypan blue dye exclusion in a Neubauer counting chamber before culturing overnight. On the next day, an aliquot of 1 × 10^5^ cells was kept for flow cytometry (input fraction). The rest of the cells were pelleted at 200× *g* for 10 min at RT and was resuspended in 60 μL MACS buffer (including PBS without Mg^2+^/Ca^2+^ + 0.5% BSA + 2mM EDTA) + 20 μL CD14 Microbeads (Milteny Biotec, #130-050-201) per 10^7^ cells. After incubation for 15 min at 4 °C, 3 mL MACS buffer was added, and the suspension was centrifuged for 10 min at 200× *g* at RT. In the meantime, the LS MACS column (Miltenyi Biotec, Bergisch Gladbach, Germany, #130-042-401) was put to the magnet and rinsed with 3 mL MACS buffer. After centrifugation, the pellets were resuspended in 500 μL MACS buffer and applied to the column, and the flow-through was collected for flow cytometry. Subsequently, the column was washed, 5 mL buffer was added, the column was removed from the magnet, and labeled cells were flushed out using the plunger (eluate fraction). To validate the isolation of CD14^+^ cells, 1 × 10^5^ cells in 80 μL stain buffer (1% BSA in PBS), flow-through, or eluate fraction were stained with 20 μL (1μg/mL) PE-conjugated CD14 antibody (Beckman, Krefeld, Germany, #A07764) or 1 μL PE-conjugated IgG2a kappa isotype control (Thermo Fisher Scientific, #12-4724-82) and kept for 30 min in the dark at RT. After adding 300 μL stain buffer, fluorescence was detected on a CytoFLEX S device and data were analyzed via FlowJo X.0.7.

### 4.8. Differentiation of CD14^+^ Primary Monocytes into M1 Macrophages

1 × 10^5^ CD14^+^ cells of the elution fraction were seeded into ThinCert cell culture inserts for 6-well plates (Greiner Bio-One, Kremsmunster, Austria) and cultured in growth medium (DMEM/F12 GlutaMAX I media supplemented with antibiotics (200 U/mL penicillin, 0.2 mg/mL streptomycin, and 2.5 μg/mL amphotericin B, all from Sigma-Aldrich)) and 10 ng/mL GM-CSF (Miltenyi Biotec, #130-093-862) for two weeks to obtain resting M0 macrophages (rM0). Media were changed every 3 days. Afterwards, rM0 were activated to the M1 phenotype by adding 20 ng/mL IFN-γ (Sigma-Aldrich Chemie GmbH) and 500 ng/mL LPS (Sigma-Aldrich Chemie GmbH) to fresh culture media for 2 days. To monitor the differentiation of monocytes, 1 × 10^5^ cells were seeded into 6-well plates, differentiated, and harvested for RNA extraction to analyze p21 and inducible nitric oxide synthase (iNOS) via RT-qPCR, as described below. In parallel to primary CD14^+^ monocytes, the THP-1 monocytic cells obtained from ATCC were also activated and differentiated into M1 macrophages, as described earlier [73].

### 4.9. Co-Cultivation of Osteoarthritic Chondrocytes and M1 Macrophages

To establish the co-culture, chondrocytes were seeded in 6-well plates (9.5 × 10^4^ cells/well) and cultured in 2 mL growth medium for 48 h, before transferring the ThinCerts bearing differentiated M1 macrophages to the chondrocyte culture plates after washing macrophages and chondrocytes once with serum-free growth medium. The co-culture is schematically presented in Figure 1A. The co-culture medium was supplemented with 10% FCS, and CPRP or hypACT blood products or EVs enriched from either blood product. The supplementations were normalized to the particle amounts present in 200 μL hypACT, which is equivalent to a 10% hypACT supplementation of growth medium. Specifically, 1.91E8 ± 1.09E8 (SD) EVs or 1.91E8 ± 1.65E8 (SD) EVs for hypACT and CPRP, respectively, of resuspended EV pellets (P100 fraction) were added to 2 mL co-culture medium. The concentration of CPRP blood product supplementation was adjusted accordingly to match the EV concentration added to the CPRP-EV-supplemented co-culture, so that the blood product would bring a similar amount of EVs as present in the EV-only-supplemented culture. After 48 h, supernatants were stored at –80 °C and RNA was extracted immediately.

### 4.10. RNA Extraction and Reverse Transcription Quantitative PCR (RT-qPCR)

Total RNA was extracted from chondrocytes cultured in the inflammation model and M1 macrophages using a High Pure RNA Isolation Kit (Roche, #11828665001). A Transcriptor cDNA Synth Kit (Roche, #04897030001) was used to synthesize cDNA according to the manufacturer’s protocol. For qPCR, FastStart Essential DNA Probes Master (Roche, #06402682001) was mixed with 1 μL cDNA and 900 nM of primer (Table 1). CT values were obtained on a LightCycler 96 device (Roche, #05815916001). Data were normalized to GAPDH, and fold changes were calculated via the ΔΔCt method.

### 4.11. Enzyme-Linked Immunosorbent Assay (ELISA)

Concentrations of IL-1β, TNF-α, and IL-6 in the supernatant of the co-culture were measured by ABTS-based ELISA assays (PeproTech, London, UK, #900-K16, #900-K25, #900-K95). Briefly, cell culture supernatants were measured undiluted or diluted 1:50 with DMEM, as required. Assays were performed according to the manufacturer’s protocol, and absorbance at 405 nm with wavelength correction at 650 nm was measured on a BioTek Synergy 2 plate reader. Concentrations of the respective cytokines were determined by BioTek Gen5 software version 1.11.5.

### 4.12. Statistical Analysis

Data were analyzed via GraphPad prism 9.0.2. Unless otherwise stated, a pairwise two-tailed *t*-test was used to compare groups. Gene expression and cytokine concentration data were tested via two-way ANOVA and Tukey’s post-hoc test in order to assess differences between treatment groups, considering a *p*-value of less than 0.05 to be significant. Multiple *t*-tests were used to test data within treatment groups, accepting a false discovery rate of 5% (*q* < 0.05) via the two-step linear step-up procedure of Benjamini, Krieger, and Yekutieli [74].

## 5. Conclusions

The described co-culture model is an accessible system that resembles an OA joint more closely than a 2D culture of chondrocytes in isolation, because the presence of activated THP1 or primary M1 macrophages can establish crosstalk between the cell types. The model was used to evaluate how treatment with blood-derived EVs functions in this context, on chondrocytes. In contrast to complete blood product supplementation of culture media, EV treatment was associated with low proinflammatory cytokine concentration in the system. With the exception of elevated ACAN or COL2 expression in response to hypACT blood products or hypACT EVs, respectively, a low quantity and quality of gene expression changes was detected in chondrocytes via RT-qPCR. This could indicate either that the EV treatment replicates the effect of the complete blood product—which would mean that the efficacy of blood products is mediated by their EVs—or that the EV concentration isolatable via UC from blood products is too low to elicit therapeutically relevant changes in gene expression in chondrocytes. However, this does not exclude the possibility that EVs are therapeutically relevant agents in blood product therapy, as their principal target tissue could be synovial tissue rather than cartilage to resolve an inflammatory phenotype of OA joints.

## Figures and Tables

**Table 1:** Table of primers.

Gene	F-Primer	R-Primer
*SOX9*	tacccgcacttgcacaac	tctcgctctcgttcagaagtc
*COL2*	gtgtcagggccaggatgt	tcccagtgtcacagacacagat
*ACAN*	cctccccttcacgtgtaaaa	gctccgcttctgtagtctgc
*MMP3*	caaaacatatttctttgtagaggacaa	ttcagctatttgcttgggaaa
*COL1*	gggattccctggacctaaag	ggaacacctcgctctccag
*GAPDH*	ctctgctcctcctgttcgac	acgaccaaatccgttgactc

## Data Availability

Not applicable.

## Supplementary Materials

Supplementary Materials are available online at https://www.mdpi.com/article/10.3390/ijms22137224/s1.
