# Supplementary material for: Effects of Extracellular Vesicles from Blood-Derived Products on Osteoarthritic Chondrocytes within an Inflammation Model
Source: Int J Mol Sci. 2021 Jul 5;22(13):7224. doi: 10.3390/ijms22137224 (PMC8267849; doi:10.3390/ijms22137224)
Supplement: Supplementary file 1 [file ijms-22-07224-s001.zip › ijms-1185850-supplementary.pdf]

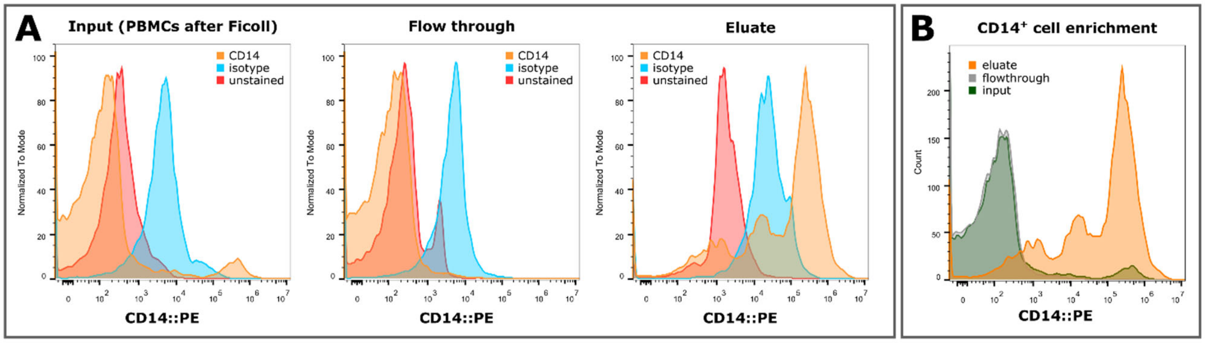

Appendix A

Figure S1. Verification of CD14$^+$ monocyte isolation via flow cytometry. (**A**) Monitoring CD14$^+$ monocyte isolation via MACS by staining input, flowthrough, and eluate fractions to assess monocyte content. While flowthrough is devoid of CD14$^+$ cells, these cells are highly enriched in the eluate fraction. (**B**) Comparison of fractions stained with CD14 antibody to highlight the enrichment of CD14$^+$ cells in the eluate fraction.
